# The role of adult romantic attachment in long-term childhood cancer survivors’ social relationships and help-seeking

**DOI:** 10.1038/s41598-026-65117-z

**Published:** 2026-08-05

**Authors:** Lea-Bianka Tröster, Hannah Marie Hermann, Manfred E. Beutel, Tamara Schwinn, Judith Hirschmiller, Jörg Wiltink, Elmar Brähler, Hiltrud Merzenich, Jörg Faber, Philipp S. Wild, Mareike Ernst

**Affiliations:** 1 Department of Psychosomatic Medicine and Psychotherapy, University Medical Center of the Johannes Gutenberg-University Mainz, Untere Zahlbacher Str. 8, 55131 Mainz, Germany; 2 University Cancer Center, University Medical Center of the Johannes Gutenberg-University Mainz, Mainz, Germany; 3 Clinic and Polyclinic for Psychosomatic Medicine and Psychotherapy, Rostock University Medical Center, Rostock, Germany; 4 Institute for Medical Biostatistics, Epidemiology and Informatics, German Childhood Cancer Registry, University Medical Center of the Johannes Gutenberg-University Mainz, Mainz, Germany; 5 Department of Pediatric Hematology/Oncology/Hemostaseology, Center for Pediatric and Adolescent Medicine, University Medical Center of the Johannes Gutenberg-University Mainz, Mainz, Germany; 6 Preventive Cardiology and Preventive Medicine, Department of Cardiology, University Medical Center of the Johannes Gutenberg-University Mainz, Mainz, Germany; 7 Center for Thrombosis and Hemostasis, University Medical Center of the Johannes Gutenberg-University Mainz, Mainz, Germany; 8 DZHK (German Center for Cardiovascular Research), Partner Site Rhine-Main, Mainz, Germany; 9 Department of Clinical Psychology, Psychotherapy and Psychoanalysis, Institute of Psychology, University of Klagenfurt, Klagenfurt Am Wörthersee, Austria

**Keywords:** Health services, Cancer

## Abstract

Childhood cancer survivors (CCS) are at risk for health-related and social challenges that can extend far into adulthood. However, CCS’ trajectories are heterogeneous. As a potential influencing factor, the present work investigated adult romantic attachment in conjunction with their relationship perceptions and experiences and help-seeking behavior. CCS (N = 633, aged 24–49 years) were recruited in cooperation with the nationwide German Childhood Cancer Registry (GCCR). Participants underwent a comprehensive medical examination at the study centre. Validated self-report questionnaires (including the ECR-RD8, PHQ-9, F-SozU K6, UCLA 3-item loneliness scale) and further items regarding help-seeking behavior were applied. Adult romantic attachment orientations were substantially related to CCS’ relationships in adulthood: Both more attachment anxiety and avoidance were linked to higher loneliness (r = 0.471, *p* < .001; r = 0.320, *p* < .001) and lower perceived social support (r = −0.349, *p* < .001; r = -0.300,* p* < .001). While 20.2% of CCS reported a need for psychosocial support, only 7.3% reported currently using a professional support offer. Lastly, attachment orientations also played a role in whether CCS voiced such a need when experiencing distress: Attachment anxiety moderated the relationship between suicidal ideation and the expression of a need for support, diminishing help-seeking. The findings emphasize the importance of integrating attachment-informed approaches in the long-term care of CCS. Tailored interventions addressing the interpersonal and emotional needs shaped by attachment styles could enhance resilience and mitigate barriers to seeking psychosocial support.

## Introduction

Childhood cancer survival rates have improved over the past few decades, transforming what was once considered a terminal diagnosis into a survivable condition for many children. The relative 5-year survival rate for pediatric cancer patients in the USA surpasses 80%^1^, and in Germany, the survival probability in the first fifteen years after diagnosis is more than 80%^2^. Therefore, childhood cancer survivors’ (CCS) long-term mental health becomes an increasingly important focus in research and practice^3^. In addition to dealing with the late effects of the disease, its treatment and developmental delays, CCS often face profound psychological and social challenges extending far into adulthood^4–6^. The long periods of hospitalization, invasive medical procedures, and physical changes associated with cancer treatment can disrupt normal childhood development and social relationships^4,6,7^, leading to delayed psychosexual development, lower rates of marriage or cohabitation, and non-independent living^4^. Young adult CCS have an elevated risk of experiencing loneliness^8^, proven to be a risk factor for anxiety and suicidal ideation^6,9^. Pahl et al.^10^ advocate for screenings for social isolation in vulnerable subgroups of CCS due to the well-established links between social connectedness and psychological health. Decades after diagnosis and treatment, CCS have a higher risk for depression, anxiety and post-traumatic stress compared to their siblings^11^ and have more medical visits for mental health complaints than the general population^12^.

At the same time, there is a substantial heterogeneity in CCS’ psychosocial outcomes, and a better understanding of the factors shaping their diverging trajectories could inform supportive measures for both current pediatric cancer patients and (long-term) survivors. Indeed, the majority of CCS do *not* suffer from clinically relevant levels of mental distress^13^. In previous studies, CCS also reported having experienced posttraumatic growth (which was related to comparatively better mental health outcomes), especially regarding close relationships^14–17^. Other positive valuations concerned CCS’ recalled parental rearing behavior, i.e., recollections of parents’ emotional support, warmth, and care throughout the disease and recovery process while growing up. These experiences were also associated with fewer depression and anxiety diagnoses^18,19^. This finding is consistent with the wealth of evidence for the sustained relevance of supportive and sensitive early relationship experiences for mental health and well-being: Originating from the foundational work of Bowlby^20^ and expanded by Ainsworth^21^, attachment theory has shown how individuals develop mental representations of attachment experiences, which shape their expectations, perceptions, and responses to behavior of others: For instance, whether others will be helpful and responsive or dismissive and rejecting if one needs to rely on them.

Bowlby^22^ further proposed that the attachment system becomes especially active in times of high stress, reflecting an innate drive to seek security and support from attachment figures, which help mitigate emotional distress and foster resilience during challenging life events. Such experiences are, over time, internalized as a secure attachment style. Insecure attachment styles of anxious and avoidant attachment, according to Brennan et al.^23^, lead to difficulties in relying on and enjoying social relationships due to exaggerated worries or by becoming compulsively self-reliant, respectively. These orientations and their relevance for well-being also apply to cancer patients^24^.

Therefore, attachment is a highly relevant dimension of CCS’ well-being in multiple ways. Within the scope of this work, we will focus on its significance as an influencing factor of *relationship perceptions and experiences* (including friendships and relationships with family members as well as romantic relationships)^25,26^ and as a disposition shaping *help-seeking behavior*^27^. The former is a relevant perspective to understand the ways in which previous relationship experiences influence those made later in life, both in terms of risk and resilience. The latter is particularly important in CCS due to a lack of structured long-term care^11,28,29^. Whether survivors receive the needed support depends on their own capacity, willingness, and attitudes. While there are recommendations for follow-up care in CCS with manifest physical late effects^30^, there are no structured programs screening their mental health. Many survivors report an unmet need for formal psychosocial support^28^ like counselling, and informal social support and relationships^31^. D’Agostino et al.^32^ explained an underutilization of mental health resources through survivors’ desire to maintain normalcy.

There is a research gap concerning both kinds of potential implications of CCS’ adult romantic attachment orientations. Therefore, the present work set out to examine adult romantic attachment in CCS in conjunction with their social life, and attitudes towards as well as views on the availability of support. As not all CCS suffer from mental distress or psychosocial difficulties and many cope well without professional support^33^, we also investigated help-seeking behavior in a more nuanced way. The regression analysis focused on suicidal ideation as one severe indicator of distress and on the explicit expression of a current need for psychosocial support as the primary help-seeking-related outcome. Specifically, we hypothesized that attachment anxiety and avoidance influence whether CCS report a need for support. While both attachment anxiety and avoidance were associated with heightened distress in previous work, we hypothesize that this is due to different reasons: Whereas overly anxious individuals are excessively dependent on others, overly avoidant individuals tend to avoid dependence altogether^34,35^. Thus, in the present study, we hypothesized attachment anxiety to strengthen the association between distress and voicing a need for support, and we hypothesized attachment avoidance to diminish it.

## Methods

### Study design and participants

The data basis for this paper consists of the register-based observational study PSYNA (Psychosocial Long-Term Effects, Health Behavior, and Prevention among Long-Term Survivors of Cancer in Childhood and Adolescence), which is an add-on of the CVSS study (Cardiac and vascular late sequelae in long-term survivors of childhood cancer)^36^.

Participants in the nationwide German Childhood Cancer Registry (GCCR) were enrolled if diagnosed with cancer between 1980 and 1990 before age 15 and treated in one of the 34 participating paediatric cancer centres. CVSS/PSYNA comprised two measurement points. The first was an examination at the study center that included highly standardized, medical examinations, a computer-assisted interview and validated questionnaires, using the platform of the Gutenberg Health Study (GHS)^37^, a large, prospective community cohort. Overall, 951 CCS took part in this assessment between 09/2013 and 02/2016^36^. The second measurement point (in which 633 CCS took part) started in early 2016 and included a phone interview as well as a questionnaire assessment. Data on attachment styles, attitudes toward psychological support and its use were assessed solely at the second measurement point. Participants gave written informed consent for study participation and data retrieval. CVSS and PSYNA are carried out in accordance with the ethical standards of the institutional research committee (approved by the ethics review committee of Rhineland-Palatinate Chamber of Physicians, nr. 837.453.13(9138-F)) and with the Declaration of Helsinki.

### Data and measures

#### Sociodemographic information

Sociodemographic information includes gender, age at study assessment, marital status, currently in a relationship, living in a shared household with a partner, and parenthood.

#### Attachment

The ECR-RD8^34^ is a short version of the original Experiences in Close Relationships—Revised (ECR-R) questionnaire^23^ that captures the extent of attachment-related anxiety and avoidance^38^. The questionnaire refers to experiences in emotionally intimate romantic relationships in general, not only to a current partnership; thus, participants without a current partner were not excluded and could answer with reference to their general experiences and expectations in romantic relationships. This follows the generic ECR-R instruction, which asks respondents to rate how they generally experience close relationships rather than only a current relationship. The self-report instrument comprises eight items with response options ranging from 1 (strongly disagree) to 7 (strongly agree). Four items each inquire about attachment-related anxiety (e.g., “I often worry that my partner will not want to stay with me”) and attachment-related avoidance (e.g., “I feel comfortable sharing my private thoughts and feelings with my partner” (coded inversely)). The ECR-RD8 was validated based on a representative population sample and showed good internal consistency (ω = 0.87 for anxiety and 0.91 for avoidance). It showed acceptable fit indices in a two-dimensional Confirmatory Factor Analysis and good convergent validity with another attachment measure, the Relationship Questionnaire^34^. In the present sample, its internal consistency was good as well (ω = 0.83 for anxiety and 0.85 for avoidance).

#### Mental health

*Suicidal ideation* was assessed as one item of the Patient Questionnaire PHQ-9: “In the last two weeks, have you been bothered by thoughts that you would be better off dead, or of hurting yourself?”. Response options range from 0 (“not at all”) to 3 (“nearly every day”). Walker et al.^39^ reported that it was indicative of suicidality as confirmed by further assessment and it was also predictive of suicide^40^. Besides a continuous approach to the frequency of suicidal thoughts^41^, it is commonly used in a binary fashion, including in the prospective community cohort whose platform was used for the present investigation. Thus, the item was dichotomized to indicate the presence versus absence of suicidal ideation, with responses of 0 coded as no suicidal ideation and responses of 1 or higher (“several days” or more) coded as suicidal ideation. This dichotomization was chosen because the item was highly skewed, with few participants endorsing suicidal ideation and sparse observations in the higher response categories. We therefore treated the item as an indicator of the presence versus absence of suicidal ideation rather than assuming a linear increase across the ordinal response options.

#### Relationship perceptions and experiences

*Social support*: To assess social support, we used the F-SozU K6 (Fragebogen zur Sozialen Unterstützung, six-item short form, German for Questionnaire on social support), an abbreviated version of the German Social Support Questionnaire (F-SozU), originally by Fydrich et al.^42^. It assesses general perceived social support (not focusing on healthcare professionals, but informal support from family and friends). Its items (e.g., “I receive a lot of understanding and comfort from others”) are answered on a scale from 1 (does not apply at all) to 5 (fully applies). It is a reliable instrument with good psychometric properties^43^ usable across the lifespan^44^. It showed acceptable internal consistency in the present sample (ω = 0.65).

*Loneliness:* We used the validated German version^45^ of the 3-item UCLA Loneliness Scale^46^ whose long form was initially developed by Russell et al.^47^. It indirectly measures loneliness, asking about experiences of feeling socially isolated, left out, and lacking company (with the response options never, rarely, sometimes, often, and very often). Klein et al.^48^ reported good psychometric properties and measurement invariance regarding age and gender based on the German general population. It showed good internal consistency in our sample (ω = 0.77).

#### Help-seeking behavior: use of and attitudes toward psychosocial support

To assess help-seeking behavior, specific items were created for the present study. They were developed to address the lack of detailed information on psychosocial support needs in long-term CCS and were informed by previous literature as well as interviews with individuals with lived and living experience. The instruction was: “Every person has a different need for support. We would like to know what support services you use and how helpful these services have been for you.” They addressed 1) a need for psychosocial support (yes/no), 2) whether participants were willing to use a psychosocial support offer if needed (yes/no), and 3) whether they currently used offers of psychosocial support (e.g., psychotherapy, psycho-oncological counselling, self-help groups) (yes/no) and 4) if yes, which type of support was being used (free-text response).

Further items asked whether 5) participants currently had a *desire* to talk to someone about mental distress in connection with their illness and 6) whether they currently had an *opportunity* to do so. Both of these items had the response options 1 = not at all, 2 = a little bit, 3 = quite, 4 = strong, 5 = very strong. Another two items centred on their current care situation: Item 7) asked whether they could convey a wish to talk to their doctor (yes/no/cannot say) and item 8) whether they would take up an offer of support if their doctor diagnosed a mental disorder such as anxiety or depression and recommended psychosocial support (yes/no/cannot say). Finally, we 9) inquired about reasons for not using support. We also explored their 10) general attitude towards psychosocial support (ranging from 0 – most negative – to 10 – most positive).

These items were not intended to constitute a psychometric scale measuring one latent construct, but to cover several clinically relevant facets of support-related experiences in an exploratory way. Thus, no total score was computed and internal consistency was not estimated.

### Statistical analysis

Analyses were performed using SPSS 29 for Windows and R version 4.3.0. Descriptive measures are reported as absolute numbers, percentages, means or standard deviations. The significance level was set at *p* < 0.05. We calculated associations of sociodemographic data and self-reported questionnaires as independent t-tests and correlation analyses. The interpretation of regression coefficients and effect sizes (partial η^2^, Cohen’s *d*) follows Cohen^49^.

**Fig. 1:**
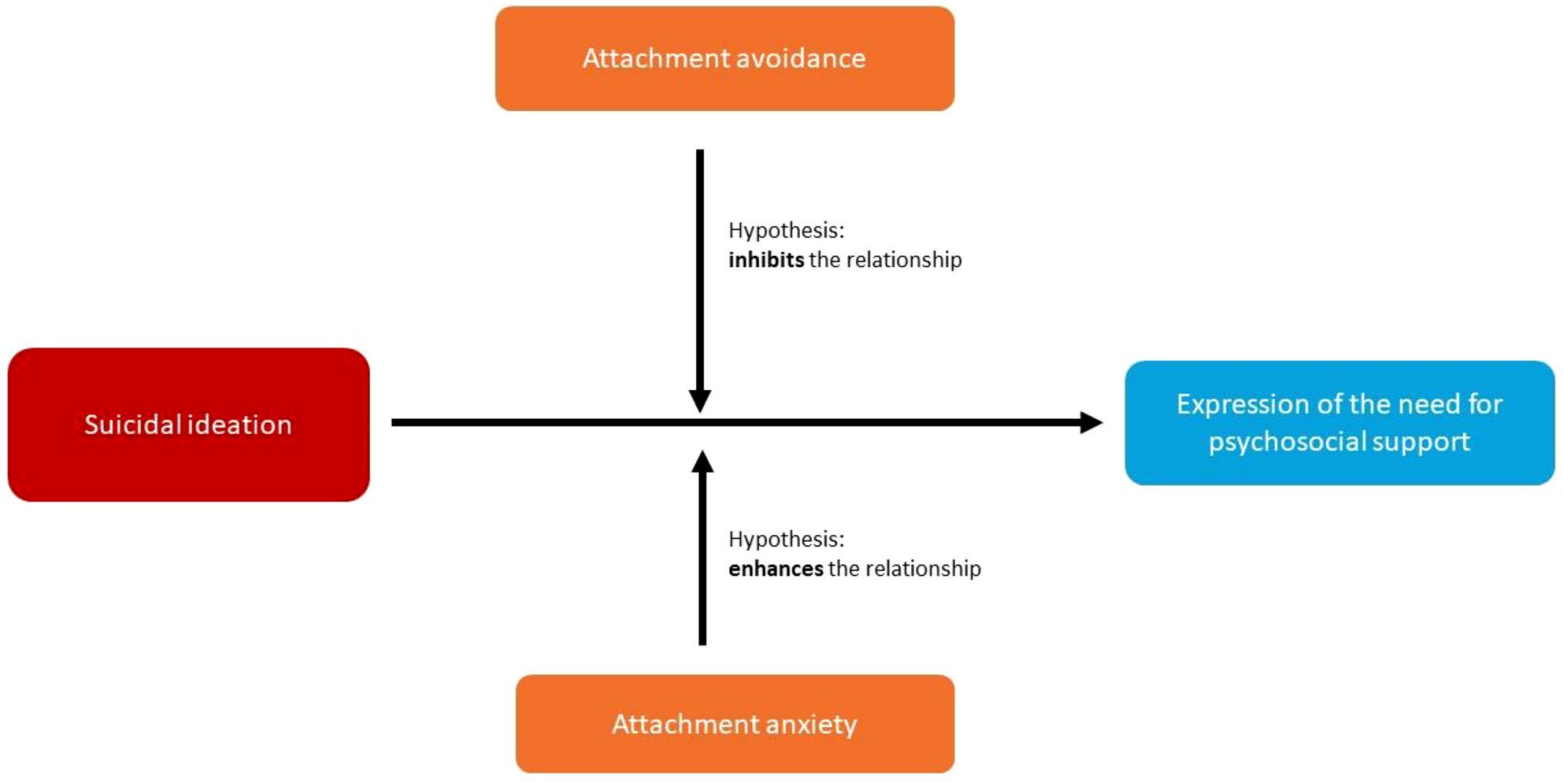
Conceptual model of the hypothesis testing using multiple logistic regression analysis.

In addition to bivariate analyses, a multiple logistic regression model was used to statistically predict the expression of the need for support as a function of suicidal ideation, attachment avoidance, attachment anxiety, and their interactions (suicidal ideation × attachment anxiety and suicidal ideation × attachment avoidance), while controlling for age and gender as covariates (Fig. 1). This item was selected as the primary outcome because it most directly captured whether CCS explicitly voiced a support need in the face of distress. Other support-related items were considered descriptive indicators of adjacent but distinct aspects, such as current service use, perceived opportunities to talk, or general attitudes toward support. We further did not compute a composite help-seeking score because the items covered conceptually distinct facets with different response formats, including perceived need, willingness, actual use, perceived opportunity, communication with physicians, and general attitudes.

The dependent variable was coded as 0 = no current need and 1 = current need. Suicidal ideation was entered as a dichotomous predictor coded as 0 = no suicidal ideation and 1 = any suicidal ideation. Continuous predictors were entered as lower-order terms on their original scales to retain the clinical and metric interpretation of the odds ratios, i.e., per one-year increase in age and per one-point increase in attachment anxiety or avoidance. For the interaction terms, mean-centered attachment anxiety and attachment avoidance scores were multiplied with suicidal ideation. Thus, the coefficient for suicidal ideation reflects its association with current need for support at average levels of attachment anxiety and avoidance. As the model included interaction terms, lower-order effects were interpreted as conditional effects and not as independent main effects. Multicollinearity was assessed using variance inflation factors and did not indicate problematic collinearity (all VIFs < 4). The regression model was based on complete cases for all model variables and missing data were not imputed.

## Results

### Sample characteristics

The sample included 633 participants. Table 1 gives an overview of their demographics. A slightly larger proportion of the sample were men. Participants’ age at the time of assessment ranged from 24 to 49 years. Their illness-related characteristics of the full sample have been reported in detail in previous publications^36^.

**Table 1:** Sociodemographic, clinical, and psychosocial characteristics of CCS (*N* = 633).

	*M* (*SD*) / *N* (%)	
Sociodemographic information		
Gender (women) (*N*, %)	281	44.4
Age at study assessment (*M*, *SD*)	34.1	5.6
Currently in a relationship (*N*, %)	428	67.6
Married (*N*, %)	253	40.7
Living in a shared household with partner (*N*, %)	422	66.8
Parenthood (*N*, %)	196	30.9
Clinical information		
Age at cancer diagnosis (*M*, *SD*)	6.34	4.38
Time since cancer diagnosis (*M*, *SD*)	28.07	3.21
Main diagnosis groups:		
Leukemias (*N*, %)	267	42.2
CNS tumors (*N*, %)	84	13.3
Lymphomas (*N*, %)	64	10.1
Others (*N*, %)	218	34.4
Attachment: ECR-RD8		
Attachment anxiety (*M*, *SD*)	2.22	1.44
Attachment avoidance (*M*, *SD*)	2.30	1.43
Suicidal ideation: PHQ-9		
Suicidal ideation PHQ-9 item 9 (*N*, %)	55	8.7
Relationship perceptions and experiences		
Social support F-SozU K6 (*M*, *SD*)	26.04	5.47
Loneliness UCLA 3-item Loneliness Scale (*M*, *SD*)	6.28	2.86

Range of the ECR-RD8 subscales: 1–7; Range of the F-SozU: 5–30; Range of the UCLA 3-item Loneliness Scale: 3–15.

### Associations of attachment anxiety and attachment avoidance with relationship perceptions and experiences

CCS without children reported higher levels of attachment anxiety than those who were parents (*M* = 2.33, *SD* = 1.49 vs. *M* = 1.99, *SD* = 1.39; *t*(425.18) = 2.81, *p* = 0.005, *d* = 0.24), but there was no difference regarding attachment avoidance. CCS living in a shared household reported lower levels of attachment avoidance (*M* = 2.13, *SD* = 1.34 vs. *M* = 2.73, *SD* = 1.56; *t*(259.37) = 4.31, *p* < 0.001, *d* = 0.43) and anxiety (*M* = 2.02, *SD* = 1.33 vs. *M* = 2.73, *SD* = 1.58; t(259.2) = 5.06, *p* < 0.001, *d* = 0.50) than those living together with others. No difference regarding attachment was found in conjunction with marital status.

Negative correlations were found between attachment avoidance (*r* = -0.300, *p* < 0.001) and attachment anxiety and social support (*r* = -0.349, *p* < 0.001). Both attachment avoidance (*r* = 0.320, *p* < 0.001) and attachment anxiety (*r* = 0.471, *p* < 0.001) were positively associated with loneliness.

### Attitudes toward and perceived availability of psychosocial support and their associations with attachment anxiety and attachment avoidance

A fifth of CCS (20.2%) reported *a need for psychosocial support*. These CCS reported more attachment anxiety (*M* = 3.07, *SD* = 1.62 vs. *M* = 2.00, *SD* = 1.31) *t*(155.8) = 6.59, *p* < 0.001, *d* = 0.78) and avoidance (*M* = 2.85, *SD* = 1.53 vs. *M* = 2.16, *SD* = 1.37; *t*(163.46) = 4.39, *p* < 0.001, *d* = 0.49) than those not reporting a need.

The 7.3% of CCS *currently using support* reported higher levels of attachment anxiety than the rest of the sample (*M* = 3.23, *SD* = 1.54 vs. *M* = 2.13, *SD* = 1.4; *t*(543) = 4.79, *p* < 0.001, *d* = 0.79), but there was no group difference regarding avoidance (*p* = 0.10). Among the reported types of support (with some CCS also reporting support used in the past), psychotherapy was the most common answer with 18 mentions. Other types comprised self-help groups, body psychotherapy, counselling, and using alternative practitioners.

Those *willing to use a support offer if needed*, a fourth of the sample (25.1%), did not report more attachment avoidance, but anxiety (*M* = 2.37, *SD* = 1.47 vs. *M* = 2.08, *SD* = 1.30, *t*(266.9) = -2.104, *p* = 0.036, *d* = 0.19), with a small effect size.

**Fig. 2:**
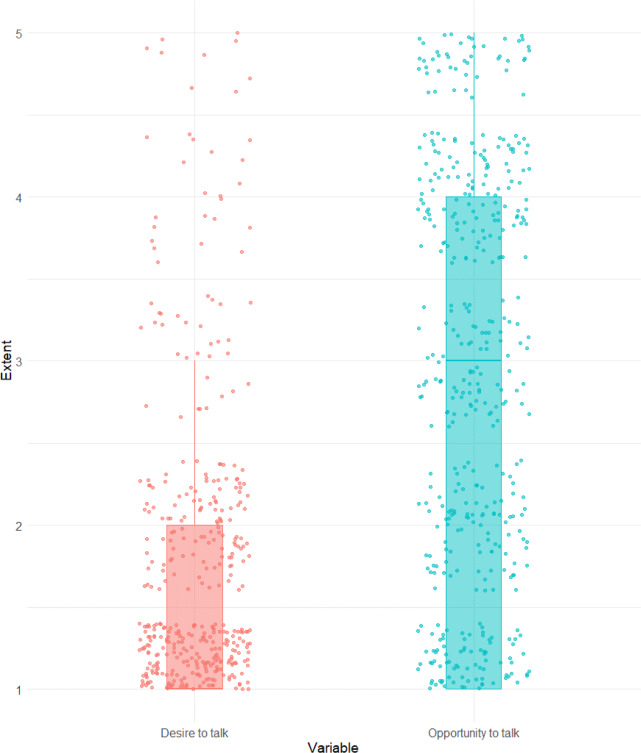
CCS’ reported desire and opportunity to talk about mental distress in connection with their illness. On the y-axis, 1 indicates very little, and 5 a lot of desire/opportunity. The boxplots visualize the distribution of values on the two items, with each box representing the interquartile range (IQR), the range between the 25th and 75th percentile and the thick horizontal line inside each box indicating the median.

Overall, the current *desire* to talk about mental distress in connection with the illness was rated as lower than the *opportunity* to talk (Fig. 2). Positive correlations were found between the *desire* to talk and attachment anxiety (*r* = 0.205, *p* < 0.001) and avoidance (*r* = 0.210, *p* < 0.001). The reported *opportunity* to talk was negatively related to both attachment anxiety (*r* = -0.122, *p* = 0.004) and attachment avoidance (*r* = -0.158, *p* < 0.001).

There was no difference between CCS who said they could and those who said they could *not* convey a wish to talk to their doctor regarding attachment anxiety (*p* = 0.29) or avoidance (*p* = 0.056). CCS reported diverse reasons for not using support: 376 (64.1% of those not using support) found it unnecessary, 51 (8.7%) did not know where to find support, and 123 (21.0%) did not know about any offers. Further reasons given were that they had never been offered support, that their healthcare provider was not/no longer paying for it, but CCS also reported feeling well and/or having enough informal support so that they did not depend on professional offers.

At the group level, CCS’ general opinion toward psychosocial support was skewed positively (*M* = 7.61, *SD* = 2.34, on a scale from 0 to 10, with higher values indicating greater appreciation). A more positive opinion was negatively associated with attachment avoidance (*r* = -0.112, *p* = 0.010). The association with attachment anxiety was not significant (*p* = 0.63).

The associations of attachment anxiety and avoidance with both relationship perceptions and experiences as well as with the dimensions of help-seeking are summarized in Table 2.

**Table 2:** Overview of associations of the variables of interest with attachment avoidance and anxiety.

	Association of…	
	Attachment avoidance	Attachment anxiety
… with relationship perceptions and experiences		
Parenthood	n. s	↓
Shared household	↓	↓
Married	n. s	n. s
Social support	↓	↓
Loneliness	↑	↑
… with attitudes toward and perceived availability of support		
Need for psychosocial support	↑	↑
Currently using support	n. s	↑
Will to use support if offered	↑	↑
Desire to talk	↑	↑
Opportunity to talk	↓	↓
Could convey a wish to talk to their doctor	n. s	n. s
General attitude toward psychosocial support	↓	n. s

Positive association; negative association; n.s. = no significant association.

### Multiple logistic regression analysis of expressing a need for support

**Fig. 3:**
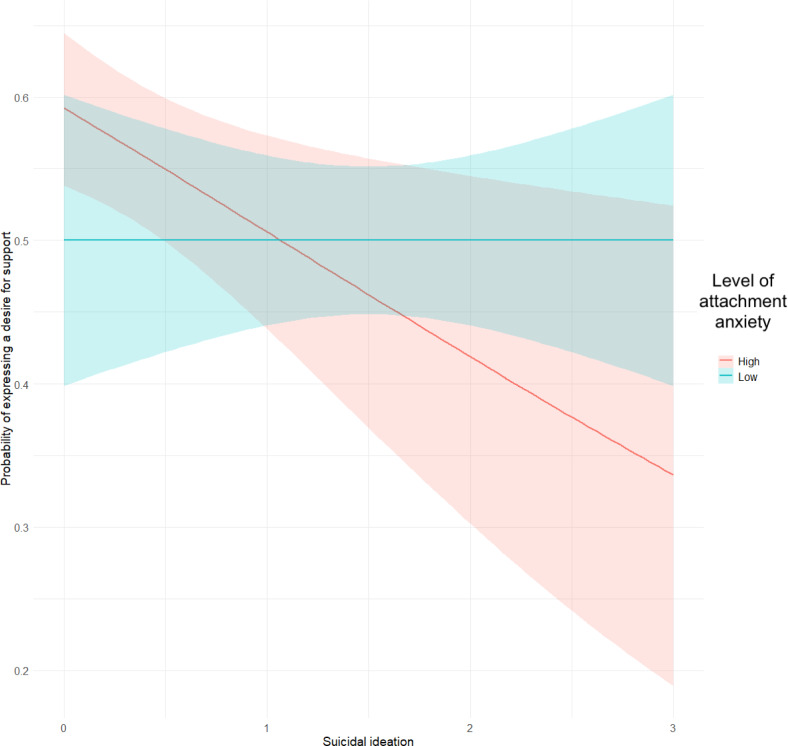
The expression of a need for support as a function of childhood cancer survivors’ suicidal ideation and comparatively low vs. high attachment anxiety. The shading indicates the 95% Confidence Interval.

Within the logistic regression model of the expression of the need for psychosocial support (Table 3), we observed effects of gender, attachment orientations, suicidal ideation, and the interaction of the latter two variables. Women were more likely than men to indicate a need for psychosocial support (OR = 2.14), as were CCS experiencing suicidal ideation (OR = 67.05), higher levels of attachment anxiety (OR = 1.47) and attachment avoidance (OR = 1.23). There was also a statistically significant interaction of suicidal ideation and attachment anxiety in the sense that individuals experiencing comparatively high levels of both were *less* likely to voice a need for support (OR = 0.56). Because the model included interaction terms, the main effect of suicidal ideation represents its association with support need at average levels of attachment anxiety and avoidance. The large odds ratio and wide confidence interval indicate a strong but imprecisely estimated association. This association is visualized in Fig. 3.

**Table 3:** Multiple logistic regression of expressing a current need for psychosocial support.

Predictors	OR (95% CI)	*p*
Intercept	0.01	< 0.001
Age	1.00 (0.96–1.04)	0.94
Gender (women)	2.14 (1.34–3.41)	0.001
Suicidal ideation	67.05 (12.49–360.10)	< 0.001
Attachment anxiety	1.47 (1.25–1.73)	< 0.001
Attachment avoidance	1.23 (1.04–1.45)	0.019
Suicidal ideation x Attachment anxiety	0.56 (0.41–0.78)	< 0.001
Suicidal ideation x Attachment avoidance	0.81 (0.61–1.07)	0.14

Nagelkerke R^2^ = 0.252. OR = Odds Ratio. CI = Confidence Interval.

## Discussion

This study investigated adult romantic attachment in relation to relationship perceptions and experiences, as well as several support-related indicators, in a sample of 633 long-term CCS. The findings highlight the importance of attachment styles in shaping CCS’ outcomes in various life domains. Although the clinical heterogeneity of CCS should be kept in mind when interpreting these findings, both attachment anxiety and attachment avoidance were relevant, yet their relative importance and specific effects varied across different domains.

A growing body of research shows adult CCS to be vulnerable^50^ to clinically relevant distress including anxiety, depression and post-traumatic stress symptoms, loneliness and social isolation^51^, and severe mental health events like late and recurrent suicidal ideation^11^, emergency department stays, hospitalization, and death by suicide^12^. The prevalence rates of anxiety, depression, and suicidal ideation within the present sample were previously shown to be higher than in the German general population^52^, with 32% of CCS reporting some form of clinically relevant distress. Along these lines, in the present study, a fifth of CCS indicated a current need for support. Unfortunately, at the same time, fewer than a tenth (7.3%) of CCS reported currently receiving psychosocial support, highlighting a substantial proportion of CCS with unfulfilled needs. This result is in accordance with expert guidelines stressing the need for ongoing mental health surveillance, and a biopsychosocial approach to long-term survivorship care^53^.

However, the variance in the sample must also be acknowledged, with most CCS *not* reporting clinically relevant levels of distress^52^. Also with respect to relationship experiences and perceptions, the present results yield a more complex picture than one of general vulnerability. If we contrast the present CCS cohort with the validation sample from the general population^34^, CCS’ levels of insecure attachment are comparatively low – potentially indicating that the relationship experiences with their parents are a resource rather than a vulnerability for CCS, in line with previous work finding posttraumatic growth especially with regard to relationships^14–16,54^. At the same time, an attachment-theoretical perspective also requires acknowledging the particular vulnerability of the parent–child dyad during childhood cancer. Diagnosis and treatment may increase parental closeness and protection, but also fear-driven overprotection and restrictions of autonomy. Previous analyses from the same registry-based cohort showed that CCS remembered both parents as emotionally warmer, more overprotective, less rejecting/punishing, and less ambitious than the general population, while more intensive treatments were associated with stronger memories of maternal warmth^55^. In turn, recalled maternal warmth was associated with fewer lifetime diagnoses of depression and anxiety, whereas paternal control/overprotection was associated with a higher likelihood of anxiety diagnoses^18^. Thus, the present findings should not be read as contradicting dyadic vulnerability. Rather, attachment-related outcomes in CCS may reflect the balance between resilience-promoting parental availability and potentially constraining adaptations to threat.

Attachment also played a significant role in CCS’ relational experiences. Survivors with higher attachment anxiety were more likely to report loneliness and reduced perceived social support, as were those with elevated attachment avoidance. By contrast, CCS living with a partner in a shared household reported lower levels of both attachment anxiety and avoidance compared to those not cohabitating. This finding highlights the far-reaching relevance of attachment styles, helping individuals to enter close, supportive relationships, particularly those characterized by physical and emotional proximity, or hindering them from it.

In terms of help-seeking behavior, both attachment styles were associated with distinct patterns of support utilization and attitudes toward psychosocial care. Survivors who expressed a need for psychosocial support or were currently using such services exhibited higher levels of attachment anxiety and avoidance, with attachment anxiety being especially pronounced. These findings are consistent with the notion that attachment anxiety is characterized by heightened emotional dysregulation and a tendency to seek reassurance, even if such behaviors are accompanied by interpersonal ambivalence^56,57^. Scheffold et al.^27^ had already found that attachment orientations influence reactions to feelings of dependency and perceptions of social support. Evidence from individuals with cancer also shows that these orientations have implications for the psychological adjustment to cancer (survival)^38^. Lastly, in our study, attachment avoidance exhibited a small negative correlation with general attitudes towards support, which is consistent with previous findings^35,57^.

We revealed an unexpected moderating role of attachment anxiety in the relationship between suicidal ideation and the expression of a need for support, in the sense that higher levels of attachment anxiety *diminished* the association. We had hypothesized attachment anxiety to strengthen the association of interest, building on previous work linking attachment anxiety to amplified distress^58^, limited capacity for flexible adaptation^24^, and more frequent primary care utilization^59^. However, contrary to our predictions, attachment anxiety *attenuated* this link. Perhaps this is because both are associated with heightened emotional dysregulation and interpersonal difficulties^60,61^, so that their combination undermines the likelihood of openly seeking help. Another interpretation could lie in conflicting emotional regulation strategies, whereby attachment anxiety drives behaviors like seeking reassurance or intensifying relational closeness^59^, whereas suicidal ideation may lead to withdrawal due to feelings of shame and guilt^62^. Also, these previous studies did not necessarily assess romantic attachment specifically. This distinction may be relevant for interpreting our finding: Romantic attachment anxiety may not simply translate into broader help-seeking, but could also involve fears of burdening or losing the partner when disclosing mental health needs. In the context of suicidal ideation, such fears may intensify interpersonal threat and make the explicit expression of support needs more difficult. Thus, the combination of suicidal ideation and high romantic attachment anxiety may inhibit, rather than facilitate, voicing a need for support. This pattern is also compatible with the concept of help-negation^63^, referring to reduced help-seeking despite increased suicidal ideation, and suggests that romantic attachment anxiety may be one interpersonal condition under which this gap becomes particularly pronounced.

We had also assumed attachment avoidance to inhibit the relationship because avoidantly attached patients express little need for help, can hardly bear to be dependent on others^64^, downplay symptoms in order to stay independent^58^ and feel hindered from seeking counselling^57^. However, we found no significant interaction effect and on its own, avoidant attachment was associated with a higher likelihood of expressing a need for support. This pattern also reflects the difference in magnitude between the main effects of attachment anxiety and attachment avoidance, in the sense that attachment anxiety appears to be even more relevant for the variance explanation in the model than attachment avoidance. This mirrors previous studies focusing on mental health outcomes^27,65,66^.

Thus, in summary, these results suggest that adult romantic attachment orientations may be relevant for understanding interpersonal experiences and the expression of support needs in long-term CCS. Rather than indicating a general pattern of help-seeking, our findings indicate that in the context of suicidal ideation, high romantic attachment anxiety may make it more difficult to explicitly voice a need for psychosocial support. Future work should examine how different facets of help-seeking—need, willingness, access, opportunity, and actual service use—are shaped by broader interpersonal and attachment-related processes. There has been increasing evidence highlighting the need for continued psychosocial support in the long-term survivorship phase^50,53^. Along these lines, Galán et al.^31^ note that adult and young cancer survivors’ most common needs were individualised information and advice, counselling, psychological and social support, a pattern well in line with the present findings among CCS.

Limitations refer to the study design, representativity and transferability. Firstly, the cross-sectional design does not allow an interpretation of the direction of associations. Further, the study relied on self-report data that could be influenced by different biases (i.e., memory, social desirability). Participant self-selection could have led to an underrepresentation of either the most vulnerable CCS for whom participation would have been an undue burden, but also to CCS coping well and not identifying as cancer survivors/not feeling burdened by their medical history. This could affect the estimation of the prevalence of mental health challenges and attachment-related barriers to support-seeking. In addition, data was collected using screening instruments and (regarding attitudes towards psychosocial support) single items, implicating a lack of contextual information regarding both CCS’ mental health (esp. regarding the questions surrounding suicidality, such as the time frame in which suicide attempts occurred) and deeper insight (e.g., if CCS reported having no opportunities to talk about mental distress, we did not know whether they are generally poorly cared for, whether they are perhaps involved with healthcare professionals with whom they do not have a good working alliance or whom they do not perceive the relevant expertise, etc.). The single items were developed to address the present research gap and drew on interviews with affected individuals, but they were not psychometrically validated. Accordingly, the findings should be interpreted as exploratory and item-specific. The logistic regression focused on the item assessing a current need for psychosocial support because it most directly reflected the expression of support need in the face of distress; however, other facets of help-seeking, such as actual service use, perceived availability, or willingness to use support, may follow different patterns. Further, the present analyses did not model diagnosis- or treatment-specific pathways. This was beyond the scope of the current study, which focused on attachment as a psychosocial dimension of relationships and help-seeking, and would have required sufficiently powered subgroup analyses. Previous analyses from the same cohort showed elevated rates of several forms of mental distress in CCS compared with the general population, with female gender and sociodemographic disadvantage as relevant correlates^52^. Other analyses suggested that disease- and treatment-related factors may be relevant for survivors’ relational memories: chemotherapy and radiotherapy were associated with stronger recalled maternal warmth, and recalled maternal warmth was linked to fewer lifetime diagnoses of depression and anxiety^18,55^. However, previous analyses found no differences in CCS’ Quality of Life between the main diagnosis groups^67^. Third, the sample may not fully represent the broader population of CCS. The sample was drawn exclusively from German CCS diagnosed and treated between 1980 and 1990. Advances in cancer treatments and psychosocial care over recent decades limit generalizability to CCS diagnosed and treated under more contemporary conditions. Several scope limitations should be kept in mind. Attachment was assessed as adult romantic attachment; although this is theoretically related to broader interpersonal expectations, the findings should not be generalized uncritically to attachment toward parents, healthcare providers, or support systems more broadly. The regression analysis focused on suicidal ideation as one severe indicator of distress and therefore does not represent mental health burden in its full breadth. Finally, suicidal ideation was dichotomized because of the skewed distribution of PHQ-9 item 9 and sparse higher response categories. This clinically meaningful approach does not capture gradations in suicidal ideation, which should be examined in larger samples. Relatedly, the large odds ratio for suicidal ideation in the interaction model had a wide confidence interval and should be interpreted as evidence of a strong association with expressed support need, but not as a precise estimate of its magnitude. Future studies with larger numbers of CCS reporting suicidal ideation should examine whether attachment-related help-seeking patterns differ according to the frequency or severity of suicidal thoughts.

## Conclusion

This study highlights the relevance of adult romantic attachment orientations for relationship perceptions and support-related experiences in long-term CCS. Romantic attachment anxiety and avoidance were associated with loneliness, perceived social support, and several indicators of attitudes toward and perceived availability of psychosocial support. The regression analysis focused specifically on suicidal ideation and the explicit expression of a current need for psychosocial support. Here, the interaction of romantic attachment anxiety and suicidal ideation revealed a complex pattern: although attachment anxiety is often linked to amplified reassurance seeking, CCS reporting high romantic attachment anxiety appeared less likely to voice a need for psychosocial support when experiencing suicidal ideation. These findings underscore the need for future research and clinical practice to consider attachment-related interpersonal concerns when addressing barriers to psychosocial support in long-term CCS.

## Data Availability

The datasets presented in this article are not readily available because the written informed consent of the study participants is not suitable for public access to the data and this concept was not approved by the local data protection officer and ethics committee. Access to data at the local database in accordance with the ethics vote is offered upon request at any time. Interested researchers make their requests to the Principal Investigators of the CVSS/PSYNA study. Requests to access the datasets should be directed to Philipp.Wild@unimedizin-mainz.
